# Mediating role of self-efficacy and cognitive flexibility in the relationship between critical thinking and positive mental health in Turkish nursing students: a cross-sectional study

**DOI:** 10.1136/bmjopen-2024-097631

**Published:** 2025-08-08

**Authors:** Eyüp Yurt, Çiğdem Müge Hayli

**Affiliations:** 1 Department of Education Science, Education Faculty, Bursa Uludağ University, Bursa, Nilüfer, Turkey; 2 Department of Nursing, Faculty of Health Sciences, Hakkari University, Hakkari, Turkey

**Keywords:** MENTAL HEALTH, Nursing research, Cognition

## Abstract

**Abstract:**

**Objectives:**

Positive mental health is crucial for nursing students, impacting their well-being and professional performance. It facilitates stress management throughout nursing education and career development. Limited research has examined the relationship between critical thinking and perceived positive mental health. This study examines the relationship between critical thinking and positive mental health, exploring the mediating effects of self-efficacy and cognitive flexibility.

**Design:**

A cross-sectional survey was used.

**Setting:**

The study was conducted in different public universities in Türkiye. The survey was administered via Google Forms, which included information about the purpose of the study and a consent form where participants declared their voluntary involvement.

**Participants:**

A random sampling method was used to recruit 464 students from various health sciences faculties at different universities, consisting of 44.4% males and 55.6% females. The participants, aged 18–25, had a mean age of 19.52 (SD=1.24).

**Primary and secondary outcome measures:**

The questionnaire included the following scales: Critical Thinking Disposition Scale, Positive Mental Health Scale, Cognitive Flexibility Scale and General Self-efficacy Scale. Descriptive analysis, correlation analysis and multiple mediation analysis were used to analyse the data.

**Results:**

The findings indicate that critical thinking is positively associated with self-efficacy (β=0.58, p<0.001), cognitive flexibility (β=0.25, p<0.001) and positive mental health (β=0.26, p<0.001). Self-efficacy also shows a positive relationship with cognitive flexibility (β=0.51, p<0.001) and positive mental health (β=0.27, p<0.001), while cognitive flexibility is positively associated with positive mental health (β=0.21, p<0.001). Multiple mediation analyses revealed that self-efficacy and cognitive flexibility accounted for 51.85% of the total effects, with self-efficacy alone explaining 29.63%. Both self-efficacy and cognitive flexibility served as sequential and parallel mediators between critical thinking and positive mental health.

**Conclusion:**

The study highlights the importance of integrating interventions that enhance self-efficacy and cognitive flexibility in nursing education programmes to promote positive mental health outcomes. These psychological resources can strengthen both critical thinking abilities and overall well-being among nursing students. The findings recommend implementing targeted training programmes in nursing education curricula and perceived positive mental health support services through problem-based learning and simulation-based education.

**STRENGTHS AND LIMITATIONS OF THIS STUDY**

- Used multiple validated scales to capture a wide array of psychological constructs.
- Applied comprehensive mediation analyses to examine complex interrelationships and pathways among self-efficacy, cognitive flexibility, critical thinking and perceived positive mental health.
- Provides actionable recommendations for enhancing nursing education programmes, emphasising the development of critical thinking, self-efficacy and cognitive flexibility.
- The study’s cross-sectional nature provides a snapshot of relationships at a single point in time, limiting the ability to infer causality.
- Reliance on self-reported data may introduce social desirability bias and response inaccuracies.

## Introduction

 Positive mental health, encompassing emotional, psychological and social well-being, directly affects the quality of life.^1 2^ While mental health is a complex, multifaceted construct that a single measure cannot fully capture, this study focuses on positive mental health perceptions as assessed through validated instruments. Recent studies highlight a concerning trend in nursing students’ psychological well-being and positive mental health indicators worldwide. Research indicates that 34–43% of nursing students experience significant psychological distress, with rates of depression and anxiety substantially higher than in the general student population.^3^ A systematic review by McCarthy *et al*^4^ found that nursing students consistently report higher levels of stress and poorer psychological well-being indicators compared with students in other healthcare disciplines, with prevalence rates of depression ranging from 27% to 51%. These mental health challenges often persist in professional practice, with 40% of early-career nurses reporting symptoms of burnout and psychological distress.^5^ In Türkiye specifically, studies indicate that up to 47% of nursing students experience moderate to severe anxiety and depression symptoms during their education.^6^ These statistics underscore the urgent need to understand and address the factors influencing nursing students’ positive mental health and psychological well-being as they directly impact their educational success and future professional effectiveness. Given these trends and the potential role of psychological resources in promoting mental health, this study aims to investigate the relationships between critical thinking, self-efficacy and cognitive flexibility as predictors of positive mental health among nursing students and examine the mediating mechanisms through which these factors influence positive mental health outcomes.

Critical thinking is one of the most important factors that can strengthen nursing students’ positive mental health and psychological resilience.^7^ Critical thinking represents a multifaceted cognitive process involving systematically evaluating information, analysing arguments and applying reasoning skills to reach well-founded conclusions.^8^ This sophisticated cognitive ability encompasses skills such as interpretation, analysis, evaluation, inference and explanation, collectively enhancing individuals’ capacity to navigate complex situations effectively.^9^ In nursing, critical thinking skills are essential for making accurate and timely decisions in patient care.^10^ Research has shown that nurses with strong critical thinking abilities demonstrate better clinical judgement, fewer medical errors and improved patient outcomes.^11 12^ Therefore, critical thinking supports clinical performance and serves as a vital psychological asset that contributes to nursing students’ mental well-being.

Self-efficacy, another crucial construct in nursing education, refers to an individual’s belief in their ability to succeed in specific situations or perform particular tasks.^13^ In nursing, self-efficacy influences students’ confidence in performing clinical procedures, managing patient care and handling challenging situations. Studies have consistently shown that nursing students with higher self-efficacy demonstrate better academic performance, increased resilience to stress and improved clinical decision-making abilities.^14^,^16^ Furthermore, self-efficacy has been identified as a significant predictor of problem-solving ability in nursing practice, with research indicating that it mediates the relationship between self-directed learning and clinical competence.^17 18^ Together, these findings highlight self-efficacy as a core psychological resource that enhances academic functioning and emotional resilience in nursing education.

Cognitive flexibility, the ability to adapt to specific situations and generate multiple solutions to problems,^19^ is particularly relevant in the dynamic healthcare environment. For nursing students, cognitive flexibility is essential for managing complex patient situations, adapting to rapidly changing clinical conditions and implementing evidence-based practice.^20^ Research has demonstrated that nurses with higher cognitive flexibility show better adaptation to workplace challenges, improved patient communication and reduced work-related stress.^21^,^23^ Moreover, cognitive flexibility has been linked to enhanced professional autonomy and better stress management among nursing professionals.^20^ Thus, cognitive flexibility is a key adaptive trait supporting effective clinical performance and sustained psychological well-being.

### Theoretical framework

The theoretical framework of this study is grounded in Bandura’s^24^ social cognitive theory and the cognitive flexibility theory proposed by Spiro *et al*.^25^ Critical thinking is conceptualised as a higher-order cognitive skill that enables individuals to evaluate situations logically and systematically.^8^ It may influence mental health directly and indirectly by shaping how individuals perceive and respond to stressors.

In this context, self-efficacy and cognitive flexibility emerge as key psychological mechanisms. Self-efficacy refers to the belief in one’s ability to successfully carry out tasks,^13^ while cognitive flexibility involves adapting to change and generating alternative solutions.^26^ The development of critical thinking is believed to enhance problem-solving success, reinforcing self-efficacy.^27^ In turn, stronger self-efficacy helps individuals reframe stressors as manageable challenges, thus promoting positive mental health. Similarly, critical thinking fosters flexible thinking patterns, enabling individuals to approach problems from different perspectives and adapt more effectively to stressful situations.^28^

Empirical evidence supports these pathways. Prior research has shown that self-efficacy mediates the relationship between academic stress and depression,^18^ and cognitive flexibility mediates the relationship between psychological resilience and mental health.^29^ Recent studies further support these mechanisms: Ahmadi Hosseiniannejad and Shariat Kiaei^30^ found that critical thinking training significantly enhanced cognitive flexibility in university students, while Arce-Saavedra and Blumen^31^ demonstrated that self-efficacy mediated the relationship between critical thinking and well-being in teacher candidates. In nursing students, critical thinking has also been found to reduce stress through self-efficacy and problem-solving skills.^32^

Based on this theoretical and empirical foundation, the present study proposes that self-efficacy and cognitive flexibility mediate between critical thinking and positive mental health. This integrated model comprehensively explains how these psychological resources jointly promote well-being in nursing students.

### Present study

Building upon the identified theoretical foundations and research gaps, the present study examines the multiple mediating roles of self-efficacy and cognitive flexibility in the relationship between critical thinking and positive mental health among Turkish nursing students. This research addresses several critical limitations in the existing literature. First, previous studies have been conducted primarily in specific cultural contexts, particularly in Eastern Asian and Middle Eastern countries, with limited representation from diverse educational and healthcare systems.^33^,^35^ Second, existing research has examined mediating variables in isolation rather than exploring their potential interactive effects through serial mediation pathways.^33 34 36^ Third, the unique combination of self-efficacy and cognitive flexibility as sequential mediators has not been investigated, despite their strong theoretical relevance to critical thinking processes and mental health outcomes.

The present study is particularly significant for nursing education and practice for several reasons. Nursing students face distinctive challenges that can substantially impact their mental health, including intense academic pressure, clinical practice stress and the emotional demands of patient care.^37^,^39^ Understanding the psychological mechanisms that promote their positive mental health and build psychological resilience is crucial for developing evidence-based educational interventions. Additionally, Türkiye’s unique position bridging Eastern and Western educational and healthcare approaches provides valuable insights into how these cognitive and psychological relationships manifest across diverse cultural contexts.

### Study hypotheses

Based on our theoretical framework and the identified research gaps, the following hypotheses were formulated.

#### Direct relationship hypotheses

- H1a: critical thinking will be significantly and positively associated with positive mental health among Turkish nursing students.
- H1b: critical thinking will be significantly and positively associated with self-efficacy among Turkish nursing students.
- H1c: critical thinking will be significantly and positively associated with cognitive flexibility among Turkish nursing students.
- H2a: self-efficacy will be significantly and positively associated with cognitive flexibility among Turkish nursing students.
- H2b: self-efficacy will be significantly and positively associated with positive mental health among Turkish nursing students.
- H3: cognitive flexibility will be significantly and positively associated with positive mental health among Turkish nursing students.

#### Mediation hypotheses

- H4a: self-efficacy will significantly mediate the relationship between critical thinking and positive mental health among Turkish nursing students.
- H4b: cognitive flexibility will significantly mediate the relationship between critical thinking and positive mental health among Turkish nursing students.
- H4c: the relationship between critical thinking and positive mental health will be significantly mediated through a serial mediation pathway involving self-efficacy and cognitive flexibility among Turkish nursing students.

**Figure 1:**
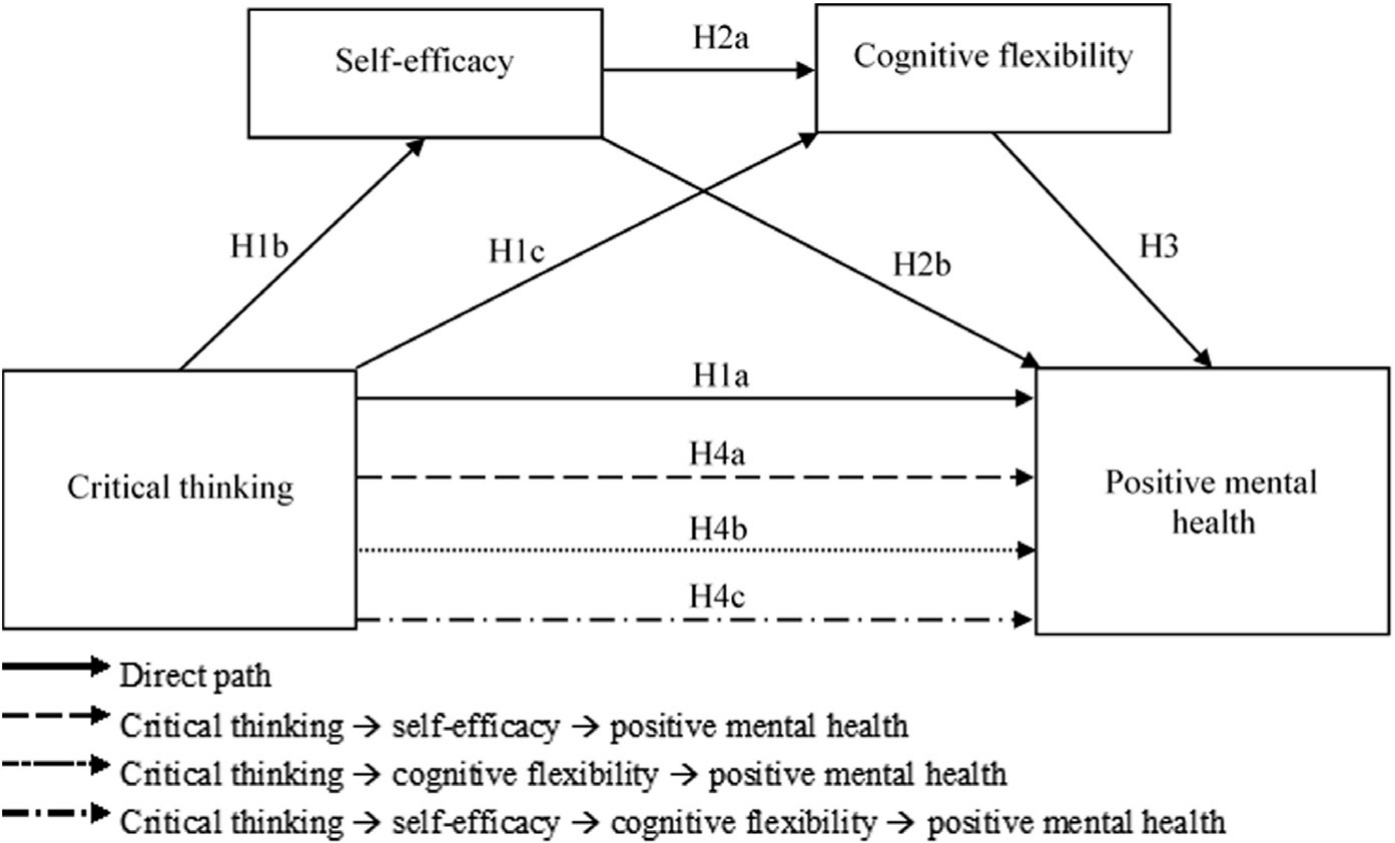
Modelling the mediating factors in the relationship between critical thinking and positive mental health. Solid lines represent direct effects, while dashed, dotted, and dash-dot lines represent indirect (mediating) effects.

These hypotheses are systematically conceptualised and visually represented in our proposed theoretical model (figure 1), providing a comprehensive framework for understanding the complex cognitive and psychological dynamics within nursing education contexts.

## Methods

### Participants and procedure

Between March and May 2024, we conducted a cross-sectional, descriptive study to explore the relationships between critical thinking disposition, cognitive flexibility and self-efficacy among nursing students from diverse health sciences faculties across Türkiye. Eligibility criteria for participants included (1) being an undergraduate student enrolled in a health sciences faculty at a public university in Türkiye, (2) having been enrolled full time for at least one academic year, (3) being able to read and understand Turkish and (4) having no diagnosed psychiatric or neurological disorders that could potentially influence mental health outcomes.

To control for factors that could influence participants’ mental health outcomes, students with diagnosed psychiatric or neurological conditions were excluded from the study. Participants were screened through the demographic information form, where they were asked whether they had received professional help for any mental health issues, were currently taking psychiatric medications or had any diagnosed psychiatric/neurological conditions.

The target population comprised approximately 10 000 students. The sample size calculation was based on the following parameters: a 95% confidence level, a 5% margin of error and an assumed medium effect size (f²= 0.15).^40^ This calculation yielded a minimum required sample size of 370 to represent the population adequately. A stratified sampling method was used ensure representation across different class levels and account for potential non-responses while ensuring a large sample size. 600 students were randomly selected from a comprehensive list of eligible students provided by the participating universities’ registrars. These students were invited to participate in the study via email, with the invitation including details about the study’s purpose, eligibility criteria and the voluntary nature of participation. All participants provided informed consent before participating in the study, acknowledging their understanding of the study’s purpose and voluntary participation.

Of the 600 invited students, 485 initially completed the survey. However, 21 students were subsequently excluded due to reported psychiatric or neurological conditions, resulting in a final sample of 464 participants and an effective response rate of approximately 77%. The 115 students who did not initially respond were contacted twice for follow-up via email over 2 weeks. Of these, 77 still did not reply to the follow-up attempts, and 38 explicitly declined participation, citing reasons such as time constraints or lack of interest.

The survey was administered via Google Forms, which included detailed information about the study and a consent form where participants declared their voluntary involvement. Each question was marked as mandatory to ensure complete data collection. The survey took approximately 20 min to complete. Using random sampling, combined with the high response rate (77%) and the sample’s representativeness across different class levels, contributes to the sample’s overall representativeness, enhancing the generalisability and validity of the study findings.

### Instruments

#### Sociodemographic information questionnaire

The sociodemographic information questionnaire included three multiple-choice questions about the gender, age and grade of nursing students.

#### Critical Thinking Disposition Scale

The Critical Thinking Disposition Scale, initially created by Sosu^41^ for university students, was translated into Turkish by Akın *et al*.^42^ This instrument includes 11 items categorised into two dimensions: Critical Openness (items 1–7) and Reflective Scepticism (items 8–11). All items are positively worded, such as “I usually try to think about the bigger picture during a discussion” from the Critical Openness dimension and “I usually check the credibility of the source of information before making judgments” from the Reflective Scepticism dimension. Respondents rate these items on a 5-point Likert scale ranging from 1 (strongly disagree) to 5 (strongly agree), with higher scores reflecting greater critical thinking disposition. The calculated alpha coefficient for the scale was 0.88, indicating a high level of reliability due to internal consistency.

#### Positive Mental Health Scale

The Positive Mental Health Scale, initially devised by Lukat *et al*^43^ for university students, was translated into Turkish by Yılmaz Akbaba and Eldeleklioğlu.^44^ This scale encompasses nine items and maintains a unidimensional structure. It employs a 4-point Likert scale, with responses ranging from 0 (not true) to 3 (true). Items are positively phrased, such as “I am in good physical and emotional condition.” Higher scores denote a greater level of perceived positive mental health. The scale’s Cronbach’s alpha coefficient was determined to be 0.83, suggesting a strong level of internal consistency.

#### Cognitive Flexibility Scale

The Cognitive Flexibility Scale, initially developed by Dennis and Wal,^26^ was translated into Turkish for university students by Sapmaz and Doğan.^45^ This scale consists of 20 items, organised into two dimensions: Alternatives (13 items) and Control (7 items). All items are positively framed; for example, the Alternatives dimension includes items like “I consider multiple options before making a decision,” while the Control dimension includes items such as “I have a hard time making decisions when faced with difficult situations.” Participants rate each item on a 5-point Likert scale ranging from 1 (not at all appropriate) to 5 (totally appropriate). The Cronbach’s alpha coefficient was found to be 0.86, indicating a good level of reliability due to internal consistency. Higher scores reflect a greater cognitive flexibility disposition.

#### General Self-efficacy Scale

To measure participants’ self-efficacy beliefs, we employed the General Self-efficacy Scale. Initially developed by Schwarzer and Jerusalem,^46^ this scale was adapted by Aypay^47^ for Turkish university students. It comprises 10 items and maintains a unidimensional structure. Respondents rate each item on a 4-point Likert scale, ranging from 1 (not at all true) to 4 (exactly true). All items are positively worded; for instance, one item reads, “I can usually handle whatever comes my way.” Higher scores indicate a stronger sense of general self-efficacy. The Cronbach’s alpha coefficient for the scale was 0.86, indicating high internal consistency.

### Data analysis

Prior to delving into the data analysis, essential assumptions were meticulously verified. Outliers were scrutinised using Cook’s distance values, following the guidelines of Tabachnick and Fidell,^48^ and it was ascertained that the data set was devoid of outliers (maximum Cook’s distance=0.08<1) and missing values. To satisfy the assumption of normal distribution, skewness and kurtosis coefficients were computed, revealing that the values (−0.93≤skewness≤−0.43, –0.71≤kurtosis≤0.36) were within the prescribed ±1 range, thereby confirming the normality.^49^ The highest variance inflation factor (VIF) calculated was 2.11, indicating the absence of multicollinearity issues among the variables as VIF values were well below the threshold of 3.^50^ Harman’s single-factor test was used to evaluate potential standard method variance bias. The results of the unrotated factor analysis showed that the first factor accounted for only 33% of the total variance, suggesting that common method bias is not a significant issue in our sample data.^51^

Descriptive statistics and Pearson correlation analysis for positive mental health, critical thinking disposition, cognitive flexibility and self-efficacy were performed. For correlation analyses, Bonferroni correction was applied to control for type I error due to multiple comparisons. The adjusted alpha level was set at p<0.003 (0.05/15 comparisons). The mediating role of self-efficacy and cognitive flexibility in the relationship between critical thinking and positive mental health was tested using Model 6 of the SPSS PROCESS macro.^52^ CIs derived from the bootstrapping method (with 5000 bootstrap samples) were used to determine the significance of total, direct and indirect effects. A 95% CI that did not include 0 indicated significant mediation effects.^52^

### Patient and public involvement

No patients were involved in this study.

## Results

### Sample characteristics

Table 1 presents the sociodemographic characteristics of the nursing students. The sample comprised 44.4% male (n=206) and 55.6% female (n=258) students. The participants ranged from 18 to 25 years, with a mean age of 19.52 (SD=1.24). Regarding academic grade levels, 25% were freshmen (n=116), 30% were sophomores (n=139), 27.6% were juniors (n=128) and 17.5% were seniors (n=81). In terms of perceived socioeconomic status (SES), the majority of students identified as middle level (80.8%, n=375), followed by low level (12.3%, n=57) and high level (6.9%, n=32).

**Table 1:** Sociodemographic information of nursing students (n=464)

Variable	N	%	M	SD	Range
Gender					
Male	206	44.4			
Female	258	55.6			
Age			19.52	1.24	18–25
Grade					
Freshman	116	25			
Sophomore	139	30			
Junior	128	27.6			
Senior	81	17.5			
Perceived socioeconomic status					
Low	57	12.3			
Middle	375	80.8			
High	32	6.9			

### Preliminary analyses

Table 2 presents all study variables’ means, SD and zero-order correlations. As anticipated, critical thinking showed a significant positive association with self-efficacy (r=0.64, p<0.003), cognitive flexibility (r=0.55, p<0.003) and perceived positive mental health (r=0.60, p<0.003). Self-efficacy was likewise positively correlated with cognitive flexibility (r=0.51, p<0.003) and perceived positive mental health (r=0.53, p<0.003). Additionally, cognitive flexibility demonstrated a significant positive relationship with perceived positive mental health (r=0.52, p<0.003). Age showed a significant positive correlation with perceived positive mental health (r=0.24, p<0.003) and critical thinking (r=0.18, p<0.003). In contrast, the correlation between age and cognitive flexibility was not significant after Bonferroni correction. Furthermore, SES was not significantly associated with the core study variables. These correlational findings support the hypothesised interconnections among variables, providing a robust foundation for examining further mediation effects.

**Table 2:** Descriptive statistics and correlations among variables

Variable	1	2	3	4	5	6	7
1. Gender	1						
2. Age	−0.04	1					
3. SES	0.07	0.09	1				
4. PMH	0.03	0.24**	0.06	1			
5. CT	0.06	0.18**	0.04	0.60**	1		
6. CF	0.05	0.09	−0.04	0.52**	0.55**	1	
7. Se	0.01	0.05	0.01	0.53**	0.64**	0.51**	1
M	–	19.52	–	1.91	3.45	3.5	2.73
SD	–	1.24	–	0.6	1	0.79	0.72
Min	0	18	1	0	1	1	1
Max	1	25	3	3	5	5	4

Gender is a dummy variable (male=0 and female=1).

**p<0.003 (Bonferroni corrected), n=464.

CF, cognitive flexibility; CT, critical thinking; PMH, positive mental health; Se, self-efficacy; SES, socioeconomic status (1=low, 2=middle and 3=high).

### Multiple mediating analysis

**Figure 2:**
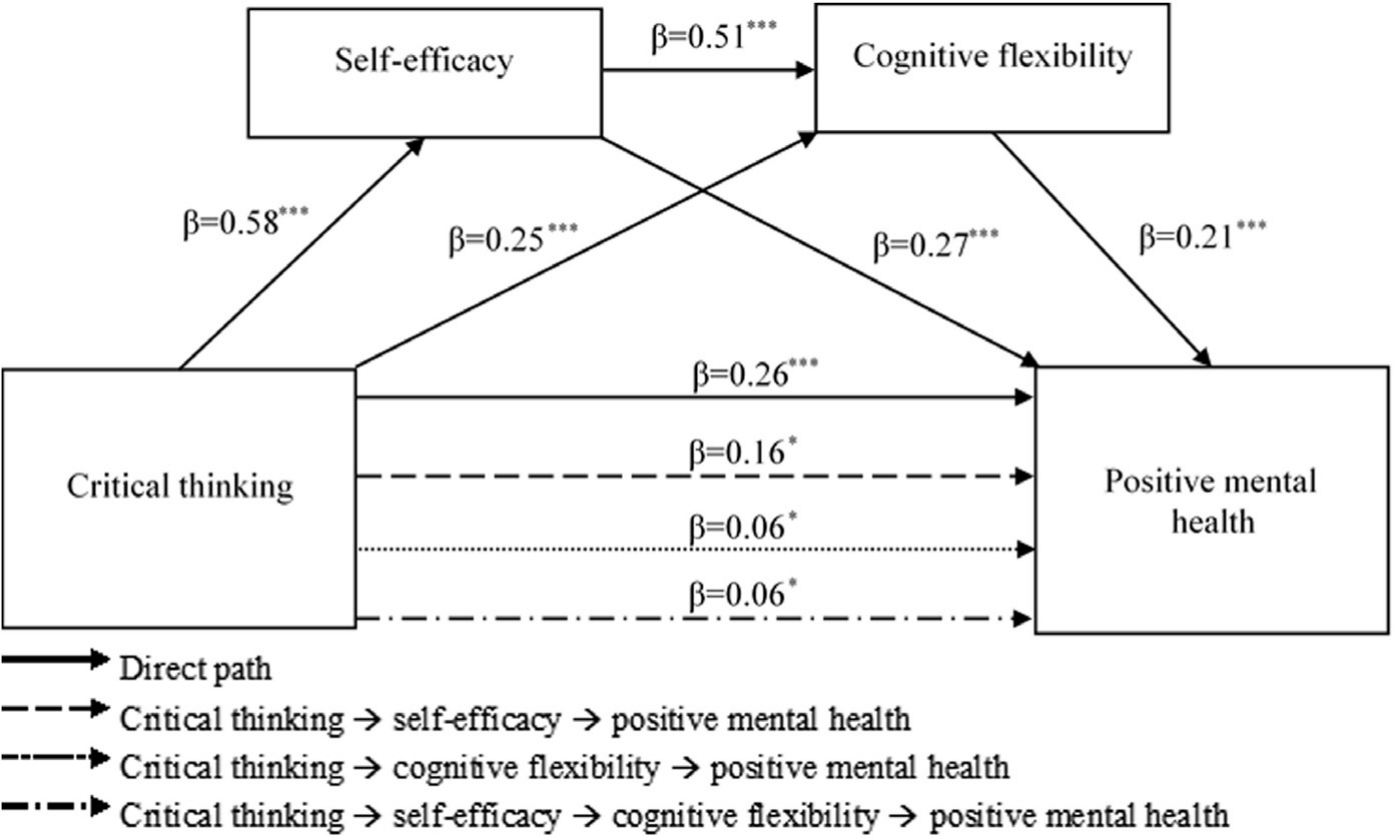
Results of the multiple mediation analysis for positive mental health, *p< 0.05, ***p<0.01. Solid lines represent direct effects, while dashed, dotted, and dash-dot lines represent indirect (mediating) effects.

Using Model 6 of the SPSS PROCESS macro, the mediating effects of self-efficacy and cognitive flexibility on the relationship between critical thinking and perceived positive mental health were tested. In the analysis, gender and age were assigned as control variables. The results of the multiple mediation analysis are presented in table 3 and figure 2. According to the standardised regression coefficients, critical thinking is positively associated with self-efficacy (β=0.58, p<0.001), cognitive flexibility (β=0.25, p<0.001) and perceived positive mental health (β=0.26, p<0.001). Self-efficacy also shows positive relationships with cognitive flexibility (β=0.51, p<0.001) and perceived positive mental health (β=0.27, p<0.001). Additionally, cognitive flexibility positively correlated with perceived positive mental health (β=0.21, p<0.001).

**Table 3:** Results of the multiple mediation analysis

Regression model outcome variable	Predictor variable	Model summary statistics			Regression coefficient and significance		Hypothesis
		R	R^*2*^	F	β	t	
Se		0.60	0.36	64.76***			
	Gender				0.05	1.38	
	Age				0.05	1.33	
	SES				0.03	0.77	
	CT				0.58	15.19***	H1b accepted
CF		0.67	0.45	75.45***			
	Gender				−0.02	−0.78	
	Age				0.10	2.80**	
	SES				0.04	1.12	
	CT				0.25	5.74***	H1c accepted
	Se				0.51	11.73***	H2a accepted
Positive mental health		0.62	0.39	48.01***			
	Gender				0.02	0.54	
	Age				0.04	0.89	
	SES				0.06	1.51	
	CT				0.26	5.46***	H1a accepted
	Se				0.27	5.11***	H2b accepted
	CF				0.21	4.09***	H3 accepted

**p<0.01, ***p<0.001.

CF, cognitive flexibility; CT, critical thinking; SES, socioeconomic status; Se, self-efficacy.

Table 4 presents the results of the bootstrap analysis. Since the calculated 95% CIs did not contain zero, all of the total, direct and indirect effects were found to be statistically significant. The multiple mediation effects accounted for 51.85% of the total effects. Specifically, the effect of the path CT → SE → PMH was calculated as 0.08, explaining 29.63% of the total effect. The effects of the paths CT → CF → PMH and CT → SE → CF → PMH were each calculated as 0.03, with each path accounting for 11.11% of the total effects. In summary, self-efficacy and cognitive flexibility served as sequential and parallel mediators in the relationship between critical thinking and perceived positive mental health.

**Table 4:** Bootstrap analysis of multiple mediation effects

Effect	Effect size (β)	SE	Percentage of total effects	95% CI		Hypothesis
				Lower	Upper	
Total effects	0.27	0.02	100	0.22	0.31	
Direct effects	0.13	0.03	48.15	0.08	0.18	
Total mediation effects	0.14	0.03	51.85	0.08	0.20	
CT → Se → PMH	0.08	0.03	29.63	0.03	0.13	H4a accepted
CT → CF → PMH	0.03	0.01	11.11	0.01	0.06	H4b accepted
CT → Se → CF → PMH	0.03	0.01	11.11	0.01	0.05	H4c accepted

N = 464, bootstrap = 5000.

→, unidirectional path; CF, cognitive flexibility; CT, critical thinking; PMH, positive mental health; Se, self-efficacy.

## Discussion

This study investigated the relationship between critical thinking and positive mental health among undergraduate nursing students, focusing on the mediating roles of self-efficacy and cognitive flexibility. Our findings from 464 undergraduate nursing students supported all hypothesised relationships and revealed significant insights into the mechanisms linking critical thinking to positive mental health outcomes.

Our findings relate specifically to positive mental health perceptions as measured by our validated scale, rather than comprehensive clinical mental health assessments. While positive mental health is an important indicator of psychological well-being, mental health is a complex, multifaceted construct that extends beyond the scope of any single measurement instrument.

First, consistent with hypothesis H1a, our results confirmed a significant positive relationship between critical thinking and positive mental health, aligning with previous research.^3336^,^39 53^ This relationship reflects the protective function of critical thinking in psychological well-being through multiple pathways: enhanced cognitive regulation, improved decision-making under stress and increased emotional awareness.^9 54^ The theoretical foundation for this relationship extends beyond cognitive behavioural frameworks to encompass metacognitive awareness and executive functioning, where critical thinking skills enable individuals to monitor and regulate their cognitive processes more effectively.^33 55^ This result underscores the importance of developing critical thinking skills for academic success and as a vital component of nursing students’ psychological well-being and professional development. Importantly, these findings should be interpreted within the context of positive mental health assessment rather than clinical mental health diagnosis.

Supporting hypotheses H1b and H1c, critical thinking demonstrated positive associations with self-efficacy and cognitive flexibility. The relationship between critical thinking and self-efficacy corroborates previous findings,^31 56^ suggesting that enhanced critical thinking abilities boost nursing students’ confidence in their capabilities. Similarly, the positive relationship between critical thinking and cognitive flexibility aligns with existing literature,^30 57^ indicating that critical thinking promotes more flexible and adaptive cognitive processes. These relationships suggest that critical thinking is a foundational skill that catalyses the development of other crucial psychological resources necessary for effective nursing practice.

In support of H2 and H3, self-efficacy and cognitive flexibility showed significant positive relationships with positive mental health. These findings are consistent with previous studies demonstrating the protective role of self-efficacy in mental health outcomes^58^,^60^ and the importance of cognitive flexibility in psychological well-being.^29 61 62^ This pattern of results highlights the crucial role of psychological resources in maintaining mental health among nursing students. It suggests that interventions targeting these factors could effectively promote psychological resilience in nursing education.

Most notably, supporting hypotheses H4a, H4b and H4c, our analysis revealed significant mediating effects of self-efficacy and cognitive flexibility in the relationship between critical thinking and positive mental health. The multiple mediation effects accounted for 51.85% of the total effects, with the path through self-efficacy (CT → SE → PMH) explaining 29.63% of the total effect, while the paths through cognitive flexibility (CT → CF → PMH) and the sequential mediation (CT → SE → CF → PMH) each accounted for 11.11% of the total effects. These findings align with Bandura’s^13 24^ social cognitive theory, suggesting that self-efficacy plays a crucial role in translating critical thinking abilities into positive mental health outcomes. The mediating role of cognitive flexibility aligns with cognitive flexibility theory,^25^ which emphasises the importance of adapting cognitive strategies and restructuring knowledge in response to changing situations—a crucial skill for maintaining mental health. The sequential mediation further indicates that critical thinking enhances self-efficacy, promoting cognitive flexibility and improving mental health outcomes. This complex mediational pathway reveals that the relationship between critical thinking and mental health is not simply direct but operates through multiple complementary psychological mechanisms.

This complex interplay suggests a synergistic relationship where critical thinking skills enhance self-efficacy and cognitive flexibility, creating a robust framework for maintaining positive mental health. The sequential mediation pathway (CT → SE → CF → PMH) particularly highlights how these constructs work together: critical thinking enhances self-efficacy, facilitating greater cognitive flexibility, ultimately contributing to better positive mental health outcomes. This finding extends previous research by demonstrating the individual mediating roles of self-efficacy and cognitive flexibility and their sequential interaction in promoting mental health. Identifying this sequential pathway provides a more nuanced understanding of how psychological resources interact to promote mental health in nursing students. While our measure provides valuable insights into positive mental health perceptions, future research should incorporate multiple indicators of psychological well-being.

These results have significant implications for nursing education and mental health practice. The strong mediating role of self-efficacy (29.63% of total effects) suggests that building confidence in one’s abilities should be a key focus alongside critical thinking development. To enhance self-efficacy among nursing students, educators can implement several evidence-based strategies: (1) providing structured clinical experiences with graduated complexity that allow students to experience success progressively, (2) incorporating peer mentoring programmes where advanced students support junior colleagues, (3) using simulation-based learning environments that provide safe spaces for skill practice and confidence building and (4) implementing reflective journaling practices that help students recognise their growth and achievements.^1363^,^65^ Similarly, the significant contribution of cognitive flexibility, both directly and as part of the sequential pathway, indicates the importance of developing adaptable thinking styles in nursing education. Specific approaches to enhance cognitive flexibility include (1) case-based learning with multiple solution pathways that encourage students to consider alternative perspectives, (2) interdisciplinary collaboration exercises that expose students to diverse viewpoints and problem-solving approaches, (3) mindfulness and cognitive restructuring techniques that help students become aware of rigid thinking patterns and (4) scenario-based training that requires rapid adaptation to changing patient conditions and unexpected situations.^2526 66^,^68^ These findings suggest that nursing education programmes adopt integrated approaches that simultaneously develop critical thinking skills while fostering self-efficacy and cognitive flexibility. Practical implementation might involve developing competency-based curricula that explicitly integrate these psychological resources, creating assessment methods that evaluate knowledge, confidence and adaptability, and establishing faculty development programmes to train educators in these holistic teaching approaches. This holistic approach to nursing education could create more resilient healthcare professionals better equipped to handle the complex challenges of modern healthcare environments.

For mental health and well-being practitioners working with nursing students, these findings offer valuable insights for intervention design and support strategies. Mental health professionals can develop targeted interventions that enhance self-efficacy and cognitive flexibility as pathways to improve mental health outcomes. Specific intervention strategies might include (1) cognitive-behavioural therapy techniques that target self-efficacy beliefs and challenge inflexible thinking patterns, (2) group therapy sessions focused on building confidence through peer support and shared experiences, (3) stress management workshops that incorporate cognitive flexibility training and adaptive coping strategies and (4) individualised counselling that helps students identify personal strengths and develop personalised resilience plans.^69^,^73^ They might implement programmes combining critical thinking exercises, confidence-building activities and cognitive flexibility training. Additionally, counselling services and mental health support programmes for nursing students could be structured to strengthen these psychological resources simultaneously, potentially leading to more effective and lasting improvements in students’ mental well-being. These practitioners could also collaborate with nursing educators to create integrated support systems that address academic development and mental health enhancement through these identified pathways.

### Limitations

Several limitations should be considered when interpreting the findings of this study. First, although the sample was randomly selected from nursing students across diverse health sciences faculties in Türkiye, the generalisability of the results to all nursing students remains limited. Future studies should include diverse samples from various universities and geographic regions to enhance representativeness. Second, the cross-sectional design provides only a snapshot of the relationships between variables at a single point in time, limiting causal inferences. Longitudinal research is needed to explore the long-term dynamics of these relationships.

Third, self-report measures may introduce social desirability bias and potential inaccuracies in participants’ responses. Future research could employ mixed-method approaches to reduce such bias and gain a more comprehensive understanding of the constructs examined. Fourth, while we statistically controlled for age, gender and SES, we did not account for other potentially influential demographic variables, which may limit the comprehensiveness of our findings.

Moreover, the Critical Thinking Disposition Scale, consisting of only 11 items, may have constrained the measurement of the full range of critical thinking dispositions, potentially limiting its content validity. Additionally, although students with diagnosed psychiatric or neurological conditions were excluded based on self-report, this method may have failed to identify undiagnosed or subclinical conditions that could impact mental health outcomes. Future studies should consider using standardised clinical screening tools to assess participants’ mental health status more accurately.

Finally, while offering valuable insights, the study’s focus on positive mental health perceptions captures only one aspect of the broader mental health construct. Future research should integrate multiple psychological well-being dimensions, including positive indicators and clinical symptoms, to provide a more holistic understanding of nursing students’ mental health.

## Conclusion

This study underscores the crucial roles of self-efficacy and cognitive flexibility in mediating the relationship between critical thinking and positive mental health among nursing students. Enhancing these skills is essential for fostering better problem-solving abilities, reducing anxiety and promoting overall well-being. Integrating critical thinking training and strategies to boost self-efficacy and cognitive flexibility into nursing education programmes can significantly improve future healthcare professionals’ mental health and professional competence. Further research should explore these relationships longitudinally and experimentally to develop effective interventions tailored to diverse educational and cultural contexts.

## Data Availability

Data are available upon reasonable request.
