# Supplementary material for: Mediating role of self-efficacy and cognitive flexibility in the relationship between critical thinking and positive mental health in Turkish nursing students: a cross-sectional study
Source: BMJ Open. 2025 Aug 8;15(8):e097631. doi: 10.1136/bmjopen-2024-097631 (PMC12336559; doi:10.1136/bmjopen-2024-097631)
Supplement: Supplementary file 1 [file bmjopen-2024-097631.reviewer_comments.pdf]

PEER REVIEW HISTORY

BMJ Open publishes all reviews undertaken for accepted manuscripts. Reviewers are asked to complete a checklist review form and are provided with free text boxes to elaborate on their assessment. These free text comments are reproduced below.

ARTICLE DETAILS

Title (Provisional)

The Mediating Role of Self-Efficacy and Cognitive Flexibility in the Relationship Between Critical Thinking and Positive Mental Health in Turkish Nursing Students: A Cross-Sectional Study

Authors

Yurt, Eyüp; HAYLI, Çiğdem Müge

VERSION 1 - REVIEW

**Reviewer**	**1**
**Name**	**Liu, Ting**
**Affiliation**	**Qingdao University**
**Date**	**31-Dec-2024**
**COI**	**None**

Overall, the manuscript is well-written with good clarity. I have some comments.

Mental health is a broad concept. In the study, the authors used positive mental health scale to measure perceived positive mental health among nursing students. Therefore, I suggest the authors to use the specific term “perceived positive mental health”, especially in the results section, including the tables and figures.

In the discussion section, the authors focused on the mechanism of the relationship between critical thinking and mental health, and highlighted the importance of self-efficacy and cognitive flexibility. To provide more valuable implications for practice, I suggest the authors to add more details regarding how to improve self-efficacy and cognitive flexibility of nursing students.

**Reviewer**	**2**
**Name**	**Filippetti, Vanessa Arán**
**Affiliation**	**Universidad Adventista del Plata, Entre Ríos, Argentina.**
**Date**	**02-Jan-2025**

COI **None**

I have received the resubmitted version of this intriguing manuscript. While the authors have satisfactorily addressed some of the reviewers' concerns, several comments have not been fully documented. I have additional suggestions regarding the current version of the manuscript.

- Since this study focuses on the construct of mental health, it is important to specify whether variables that could influence outcomes, such as neurological or psychiatric disorders, were controlled for in the study sample.

- In the Data Analysis section, please provide information about the fit indices used to test the fit of the Path models.

- For the correlation analyses, was any correction performed for multiple comparisons?

- Since SES is a variable that can influence the results, it is recommended that we control for it in path analysis.

- Throughout the discussion and manuscript, it is suggested that some statements, especially regarding mental health, be softened, considering that it is a difficult construct to evaluate. It is challenging to think that a single scale can provide a reliable indicator of mental health.

Reviewer **3**

Name **Alshowkan, Amira**

Affiliation **Imam Abdulrahman Bin Faisal University, Dammam, Saudi Arabia.**

Date **16-Jan-2025**

COI **None**

Dear Author,

Thank you for your great effort in studying such important variables among nursing students.

I Have some comments.

1. Kindly revise references to be less than 10 years old unless using classic references.

2. Study Framework, it is quite long, please summarize it.

3. The present study section, seems like a literature review, please revise.

4. Please mention the study hypothesis as a list.

Good luck

**Reviewer**	**4**
**Name**	**Abd Elhay , Eman Sameh**
**Affiliation**	**Mansoura University**
**Date**	**30-Jun-2025**
**COI**	**None**

Thank you for allowing me to review the article. I have read the manuscript submitted to the journal and I am interested. The manuscript is well-structured, it addresses a clear research gap by exploring the mediating mechanisms linking critical thinking to mental health—a topic of both theoretical and practical significance.

methods

The Critical Thinking Disposition Scale has only 11 items. Could this limit content validity? Consider discussing in limitations.

results

(Figure 1): The visual model should clearly show the sequential vs. parallel paths for easier understanding by readers unfamiliar with mediation models.

Some content in the Introduction and Discussion is slightly repetitive (e.g., explaining the link between critical thinking and mental health).

Language and Grammar: a few awkward phrases in the abstract (e.g., "a healthy mind aids in managing stress") could be revised for clarity and conciseness

VERSION 1 - AUTHOR RESPONSE

	**Reviewer requests**	**Revisions made and page number**<br><br>(The revisions have been highlighted in red throughout the manuscript.)
**Reviewer 1**	Mental health is a broad concept. In the study, the authors used positive mental health scale to measure perceived positive mental health among nursing students. Therefore, I suggest the authors to use	Thank you for this important clarification regarding the terminology used in our study. We acknowledge that mental health is indeed a broad concept, and we agree that specificity is crucial for clarity and accuracy.<br><br>We have carefully revised the manuscript to consistently use the specific term "perceived

	the specific term "perceived positive mental health", especially in the results section, including the tables and figures.	positive mental health" and "positive mental health" throughout the results section, including all tables and figures, as suggested. This revision better reflects the construct measured by the positive mental health scale and enhances the precision of our reporting.
**Reviewer 1**	In the discussion section, the authors focused on the mechanism of the relationship between critical thinking and mental health, and highlighted the importance of self-efficacy and cognitive flexibility. To provide more valuable implications for practice, I suggest the authors to add more details regarding how to improve self-efficacy and cognitive flexibility of nursing students.	Thank you for your valuable suggestion to provide more practical implications regarding how to improve self-efficacy and cognitive flexibility among nursing students. We have substantially revised the discussion section to include detailed, evidence-based strategies for enhancing these psychological resources.<br><br>The revised discussion now includes specific approaches for improving self-efficacy (such as structured clinical experiences, peer mentoring, simulation-based learning, and reflective practices) and cognitive flexibility (including case-based learning, interdisciplinary collaboration, mindfulness techniques, and scenario-based training). We have also added practical implementation strategies for nursing education programs and mental health interventions.<br><br>These additions provide concrete guidance for educators and practitioners seeking to apply our findings in practice, making the manuscript more valuable for the nursing education community.
**Reviewer 2**	-Since this study focuses on the construct of mental health, it is important to specify whether variables that could influence outcomes, such as neurological or psychiatric disorders, were controlled for in the study sample.	Thank you for your valuable feedback regarding the control of variables that could influence mental health outcomes. We have addressed your concern by implementing the following changes to our manuscript:<br><br>Method Section (Participants and Procedure): We have added eligibility criterion (4) stating that participants with diagnosed psychiatric or neurological disorders were excluded from the study. We have also included a detailed explanation of how participants were screened through the demographic information form regarding their mental health history, professional help received, and current psychiatric medication use. Additionally, we have updated the participant flow to reflect that 21 students were excluded due to reported

		psychiatric or neurological conditions, resulting in our final sample of 464 participants.<br><br>Limitations Section:<br>We have revised the limitations to acknowledge that while we excluded students with diagnosed conditions based on self-report data, the reliance on self-disclosure may have limitations. We have noted that subclinical symptoms or undiagnosed conditions were not systematically assessed and suggested that future research should incorporate standardized clinical screening instruments for more comprehensive evaluation.<br>We believe these modifications adequately address your concern while maintaining transparency about the study's methodology and limitations.<br><br>pp. 5 and 11
**Reviewer 2**	- In the Data Analysis section, please provide information about the fit indices used to test the fit of the Path models.	Thank you for your valuable comment. In this study, the mediation analysis was conducted using Model 6 of the PROCESS macro developed by Hayes (2013), which is based on ordinary least squares (OLS) regression. As such, this method does not generate structural equation modeling (SEM) fit indices such as RMSEA, CFI, or TLI. To avoid confusion, we have revised the column heading in Table 3 from "Goodness-of-Fit Indices" to "Model Summary Statistics", which more accurately reflects the reported values (R, R^2, and F) derived from OLS regression models.<br><br>pp. 5-6
**Reviewer 2**	-For the correlation analyses, was any correction performed for multiple comparisons?	Thank you for your important question regarding multiple comparison corrections in our correlation analyses. We have addressed this concern by implementing the following changes:<br>Method Section (Data Analysis):<br><br>We have added a statement indicating that Bonferroni correction was applied to control for Type I error due to multiple comparisons, with the adjusted alpha level set at $p < 0.003$ (0.05/15 comparisons).<br><br>Table 2 (Correlations):

		We have updated the correlation table to reflect the Bonferroni-corrected significance levels. The age-cognitive flexibility correlation, which was previously significant at $p < 0.05$, is no longer significant after correction. All other correlations remain significant at the corrected alpha level of $p < 0.003$. The table footnote has been updated to reflect the Bonferroni correction.<br>We appreciate your attention to this methodological detail, as it strengthens the reliability of our correlation findings.<br><br>pp.7
**Reviewer 2**	- Since SES is a variable that can influence the results, it is recommended that we control for it in path analysis.	In response to the reviewer's recommendation to control for Socio-economic status (SES), we have included this variable in our analyses. The updated descriptive statistics and correlations are presented in Table 2, and the mediation analysis results controlling for SES are shown in Table 3.<br><br>pp.7-8
**Reviewer 2**	- Throughout the discussion and manuscript, it is suggested that some statements, especially regarding mental health, be softened, considering that it is a difficult construct to evaluate. It is challenging to think that a single scale can provide a reliable indicator of mental health.	We appreciate the reviewer's thoughtful concern regarding the complexity of mental health assessment. We acknowledge that mental health is indeed a multifaceted construct that cannot be fully captured by a single scale.<br>In response to this valuable feedback, we have carefully revised our manuscript, particularly in the Introduction, Results, Discussion and Limitations sections, to use the more precise term “positive mental health” instead of the broader term “mental health.” This revision better reflects the specific construct measured by our scale, which assesses perceived positive mental health rather than comprehensive mental health status.<br><br>We have also added clarifying statements throughout the manuscript to acknowledge the limitations of our measurement approach and to emphasize that our findings relate specifically to positive mental health perceptions rather than clinical mental health diagnoses or comprehensive mental health assessments. (eg., “These findings should be interpreted within the

		context of positive mental health assessment rather than clinical mental health diagnosis.”).<br><br>We believe these revisions provide greater precision in our terminology and better align our claims with the scope of our measurement tool.
**Reviewer 3**	Kindly revise references to be less than 10 years old unless using classic references.	Thank you for your helpful suggestion. In response, we have removed ten outdated references and incorporated 20 recent studies published within the last five years to enhance the currency and relevance of the literature. Only a few classic references have been retained where conceptually essential.
**Reviewer 3**	Study Framework, it is quite long, please summarize it.	Thank you for this valuable feedback. We have revised the theoretical framework to enhance clarity and readability while maintaining the essential theoretical foundation required for our complex mediation model. The framework has been restructured with shorter paragraphs, clearer transitions, and more concise explanations of the relationships among variables. We have also incorporated additional recent empirical evidence (Ahmadi Hosseiniannejad & Shariat Kiaei, 2023; Arce-Saavedra & Blumen, 2022; Mertayasa et al., 2024) to strengthen the theoretical rationale for our serial mediation hypothesis. The revised framework now provides a more streamlined yet comprehensive understanding of how critical thinking, self-efficacy, and cognitive flexibility interact to influence mental health outcomes in nursing students.<br><br>pp. 3
**Reviewer 3**	The present study section, seems like a literature review, please revise.	We appreciate this valuable feedback. We have revised the Present Study section to focus on the study's specific aims, significance, and unique contributions rather than providing a detailed literature review. The section now emphasizes the research gaps our study addresses and clearly articulates why this research is important for nursing education, while maintaining essential citations to support our rationale.<br><br>pp. 4
**Reviewer 3**	Please mention the study hypothesis as a list.	Thank you for this suggestion. We have restructured the hypotheses section to present all study hypotheses in a clear, organized list

		format. The hypotheses are now categorized under "Direct Relationship Hypotheses" and "Mediation Hypotheses" for better clarity and easier reference throughout the manuscript.<br><br>pp. 4
**Reviewer 4**	-The Critical Thinking Disposition Scale has only 11 items. Could this limit content validity? Consider discussing in limitations.	Thank you for this valuable suggestion. We have added a discussion of the potential content validity limitation related to the Critical Thinking Disposition Scale's limited number of items in the limitations section. This addition acknowledges that fewer items might not fully capture the breadth and complexity of critical thinking dispositions.<br><br>pp. 11
**Reviewer 4**	- (Figure 1): The visual model should clearly show the sequential vs. parallel paths for easier understanding by readers unfamiliar with mediation models.	Thank you very much for your valuable suggestion regarding the clarity of the visual model (Figure 1). In response to your comment, we have revised the figure to better illustrate both the parallel and sequential mediation paths. The updated figure now includes clearly labeled direct and indirect paths with different line styles and explanatory notes to enhance readability and understanding for readers unfamiliar with mediation models.<br><br>We greatly appreciate your insightful feedback, which has significantly improved the clarity of our manuscript.
**Reviewer 4**	- Some content in the Introduction and Discussion is slightly repetitive (e.g., explaining the link between critical thinking and mental health).	We appreciate the reviewer's observation regarding the repetitive content between the Introduction and Discussion sections, particularly in explaining the link between critical thinking and mental health. We agree with this assessment and have revised both sections to eliminate redundancy while maintaining theoretical coherence.<br>In the revised version, we have:<br><br>-Expanded the critical thinking definition in the Introduction to emphasize its multifaceted cognitive nature<br>-Removed the repetitive CBT framework explanation from the Discussion

		-Enhanced the Discussion with additional theoretical perspectives (metacognitive awareness and executive functioning)<br>-Streamlined the explanation of protective mechanisms to avoid duplication<br><br>pp.2-3 and 8-10
**Reviewer 4**	- Language and Grammar: a few awkward phrases in the abstract (e.g., "a healthy mind aids in managing stress") could be revised for clarity and conciseness	We thank the reviewer for this valuable feedback. We have carefully revised the abstract to improve clarity and conciseness. Specifically, we have rewritten awkward phrases such as "a healthy mind aids in managing stress" and similar constructions throughout the abstract. The revised version now uses more direct and academically appropriate language while maintaining the original meaning and key findings of the study.<br><br>pp.1
